# Much More Than PTSD: Mothers’ Narratives of the Impact of Trauma on Child Survivors and Their Families

**DOI:** 10.1007/s10591-017-9408-z

**Published:** 2017-04-01

**Authors:** Stephen Coulter, Suzanne Mooney

**Affiliations:** School of Social Sciences, Education and Social Work, Queens University Belfast, Belfast, UK

**Keywords:** PTSD, Psychological trauma, Narrative interviewing, Trauma impact, Mothers, Family

## Abstract

This study examined the narratives of ten Caucasian mothers whose children had been impacted by ‘traumatic’ events and referred to a specialist trauma service in N. Ireland. The research question was whether the PTSD construct adequately represented the broad ‘lived’ experience of the impact of trauma on survivors’ wellbeing and their family relationships as articulated by mothers post trauma. Narrative Interviewing methodology was employed and the resulting data inductively organised into an evolving thematic framework. A quantitative analysis of the prevalence of particular themes is presented supplemented by qualitative quotations to illustrate the complexity of reported impact. The major components of the mothers’ narratives included family and relational distress (35.7%), non-pathological individual distress (24.4%), resilience (16.7%) and a prior history of adversity (16.6%). Prior history of adversity was resent in 8 out the 10 cases including a high level of suicide. PTSD symptomatology constituted a small proportion of the narratives (6.6%) and this suggests that the PTSD construct does not adequately represent the broad ‘lived’ experience of the impact of trauma. Although a small and heterogeneous study sample, the findings are sufficiently robust to suggest further investigation is required to understand the phenomenological experience of trauma of child victims/survivors and their families.

## Introduction

It has been argued that the experience of trauma has been over-medicalised and reduced to a relatively narrow set of ‘symptoms’ associated with the diagnostic category post-traumatic stress disorder (PTSD) (van der Kolk 2002; Kleinman 2006). This pathological conceptualisation is thought to strongly influence mental health interventions with trauma victims/survivors and has the potential to obscure important dimensions of the phenomenological experience of trauma that are significant to the survivors and their families (Woodward and Joseph 2003; Blackwell 2005).

During a decade’s work at a regional trauma centre it has been the first author’s experience that clients talk about a wide variety of impacts following traumatic events. After an electronic literature search identified a very limited body of relevant research, this small scale study was developed to explore whether this clinical wisdom could be empirically supported via the rigorous application of Narrative Interviewing methodology. The research question was whether the PTSD construct adequately represented the broad ‘lived’ experience of the impact of trauma on survivors’ wellbeing and their family relationships as articulated by mothers post trauma.

The focus on mothers’ narratives was taken as a starting point as mothers are most often the child’s primary support person in the context of trauma (Van Roy et al. 2010) and the family member most likely to attend initial assessments (Coulter 2011). The site for the study was a regional trauma treatment centre in Northern Ireland, which receives referrals for children and young people under the age of 18 years affected by a wide range of traumatic events. The authors are both qualified Social Workers and Systemic Psychotherapists with broad practice experience currently working as university lecturers.

## Literature Review

Traumatic stress is the natural psycho-biological reaction to a potentially traumatising event. When stress responses persist beyond the peri-traumatic period, it is termed post-traumatic stress (PTS). The spectrum of PTS responses includes the following mental health diagnoses: anxiety disorders, post-traumatic stress syndrome, PTSD, ‘complex’ PTSD, eating disorders and dissociative disorders (Horowitz 1997). In practice it is unusual for any one of these disorders to occur in isolation with co-morbidity recognised as the norm (Kessler et al. 1995; McFarlane and Papay 1992) involving complex inter-relationships between symptoms (Brown et al. 2001). PTS responses are also known to significantly impact social, educational, occupational and family functioning leading Van der Kolk and McFarlane’s to assert that *‘focussing solely on PTSD to describe what victims suffer does not do justice to the complexity of what actually ails them’* (1996, p.16). Despite the complexities of trauma response PTSD has come to be considered the ‘*signature disorder’* in survivors of traumatic events (Breslau 2002) and the vast majority of trauma research has been conducted in relation to this disorder (Santiago et al. 2013).

The PTSD construct itself has not been free from criticism including whether it exists apart from mental health discourse (Summerfield 2001) and its failure to encompass the full breadth of psychological responses to trauma (Herman 1992; Becker 1995) and cultural variation (Tummala-Narra 2007). The definition has also been critiqued as too wide (Friedman 2013) leading to a tightening of the criteria in the most recent version of the Diagnostic and Statistical Manual of Mental Disorders (5th Ed.; DSM-5 American Psychiatric Association 2013). PTSD as a construct has nonetheless survived these critiques and remains highly influential in trauma recovery discourse and practice.

Cognitive Behavioural approaches targeting PTSD symptoms are the most commonly recommended professional intervention for PTS responses (Ehlers and Clark 2000; Iverson et al. 2011). However, this is far from the whole picture with emerging streams of literature that emphasise the need to rebuild a coherent sense of identity through narrative methods (Crossley 2000: White, 2004) and the importance of integrating the trauma memory through use of body-focused methods (Van Der Kolk 2014).

Another focus of research is the post-trauma recovery environment provided by family, friends and the wider community, which is known to reduce the intensity of PTS responses (Coker et al. 2002). Following an evaluation of the research evidence Briere and Scott propose that, *‘social support is one of the most powerful determinants of the ultimate effects of trauma’* (2006, p. 17). The detrimental impact on family relationships is widely accepted including affective dysregulation, reduced communication satisfaction, poorer role maintenance and role ambiguity, and higher rates of separation and divorce (Beckerman 2004; Henry et al. 2011). Saltzman et al. (2009) state:

*The dis-synchronicity of family members’ recovery from trauma or loss may then result in heightened levels of stress and conflict within the family. The result is compromised family support and cohesion just when these qualities are most needed to facilitate recovery* (2009:241).

Consequently, a family approach to treatment of psychological trauma is advocated by a wide range of authors (Catherall 1999; Dyregrov 2001; Walsh 2016) who argue that such approaches help integrate the traumatic experience and reactivate the natural healing processes within the family.

In short, there is concern about the extent to which the PTSD construct dominates academic thinking and research activity in relation to psychological responses to trauma. This study therefore sought to empirically test whether the PTSD construct adequately represented the ‘lived’ experience of the impact of trauma as articulated by mothers during initial post-trauma assessments.

## Method

This pilot study adopted a mixed methods design, initially utilising the qualitative approach of ‘Narrative Interviewing’ (Jovchelovitch and Bauer 2000) followed by a rigorous quantitative analysis of emerging themes. ‘Narrative Interviewing’ is argued to place more control with the informant than the question-response interview by encouraging the informant to choose his/her own wording, ordering and ‘meaning making’.

During the initial assessment interview the mother was asked the following standard single question aimed at inducing narrative(s) (SQUIN) question:

*Please tell me your story of how* [the traumatic event] *has affected you and your family?*

This question was asked by the first author, as soon as practicable following the social engagement phase of the session. The mother’s response to the SQUIN question was actively listened to without interruption. She was then invited to expand on aspects of her account with the use of prompts relating only to comments about the impact on an individual or family dynamics already mentioned. In practice, prompts were a relatively minor feature at the end of the interviews.

The study sample of mothers was recruited from families in which the mother attended the family’s initial appointment at the regional Trauma Centre. It was a convenience sample with mothers from each eligible family during the 5 month study recruitment period invited to participate. The initial appointment confirmation letter advised mothers that they would be asked for permission to audio-record the session, and at the end of the session those who agreed would be invited to give consent for the use of the recording for this study. Other family members were present during these initial appointments in all bar two interviews (see Table 1). In these cases the other family members’ contributions were transcribed but not included in the analysis. The audio-recordings were downloaded to a password protected computer, transcribed by a professional transcription service and then stored in a locked filing cabinet at Queens University Belfast after which the original recordings were erased. The study clinic received approximately 25% of the Centre’s cases during the study period. Two mothers declined to participate. The range of cases was typical for the service.

**Table 1:** Mothers’ narratives of trauma—nature/range of cases

No.	Nature of traumatic incident	Referred victim	Age at referral	Time since event	Ethnicity	Prior history of adversity	Interview attended by
1	Relatives’ house shot at by gunmen as part of a feud. Shooting witnessed by daughter	Daughter	10	5 months	White—Irish Traveller	Daughter is aware of a previous incident of a malicious fire at another house nearby	Mother and daughter
2	Overdose by teenage daughter	Daughter	13	2 months	White—British/ Irish	Multiple suicide attempts by older teenage daughter; Father in prison. History of domestic violence from father to mother	Mother
3	Rape of teenage daughter	Daughter	17	24 months	White—British/Irish	Mother suffered sexual assault previously	Mother, father and daughter
4	Teenage daughter’s mental health deterioration in context of brother’s suicide	Daughter	13	21 months	White—British/Irish	Son who died had a history of overdoses	Mother and daughter
5	Physical assault of teenage son in school	Son	16	5 months	White—British/Irish	Cousin took his own life several years ago	Mother, father and son
6	Teenage girl self-harming and displaying difficult, angry behaviour in recent months following being told the identity of her father	Daughter	16	Ongoing	White—British/Irish	Father died by suicide when daughter was a toddler. History of domestic violence, witnessed by daughter	Mother and three children
7	Threat to Father by gunman who came to home witnessed by mother and son	Son	9	3 months	White—British/Irish	Maternal grandmother took her own life about a year earlier	Mother and two children
8	Sexual and physical assault of young adult daughter	Daughter	21	12 months	White—British/Irish	Teenage son took his own life a few years ago. Another son was in a road traffic accident about a year before	Mother and father
9	Physical assault on Father, witnessed by teenage daughter	Daughter	14	10 months	White—British/Irish	None reported	Mother
10	Teenage son assaulted. Motivation was sectarian	Son	16	6 months	White—British/Irish	None reported	Mother, father and son

## Analysis

A systematic thematic analysis was undertaken by the first author and a research assistant (the second author) as follows. Full interview texts were initially read by each author to ensure understanding of the issues that emerged in the flow of conversation. The mother’s text was then extracted from the full interview and read and re-read to gain as full an understanding as possible of the content of each sentence. A sequentially numbered list of content items was created independently by each author and following rigorous discussion of their respective initial content analyses, an agreed list of content items for each case was established.

Agreed content items were independently arranged into sub-themes and themes. A ‘miscellaneous’ category was included to avoid the need to omit any content items. The allocation of content items to sub-themes, and of sub-themes to emerging themes for each case was discussed by the two authors and definitions for the subthemes and themes negotiated and agreed. Each case content item was then independently revisited and reallocated to these agreed themes, amending the initial attributions when necessary, and the authors conferred again regarding this revised thematic allocation, negotiating any discrepancies. The results for each case were combined into an over-arching analytical framework of supra-themes. This method is consistent with that recommended by Jovchelovitch and Bauer (2000, p. 70) who state, “*Text units are progressively reduced in two or three rounds of serial paraphrasing…Out of paraphrasing, a category system is developed with which all texts may ultimately be coded … A final category system can only be stabilized through iterating revisions.”*

Congruent with the study rationale and the chosen thematic analysis process, which lends itself to content analysis (Bauer 2000), a quasi-quantitative measure was created by using the number of content items allocated to each sub-theme, theme and supra-theme, to give an indication of the extent to which mothers talked about the various themes during the initial interview. Given the small sample and the different types of traumatic event represented, the data was ‘edited’ prior to analysis by excluding sub-themes that only emerged from one case in order to reduce the risk of undue influence due to the idiosyncratic circumstances of any particular case.

### Ethical Issues

The recruitment process presented the ethical dilemma of how to ensure informed consent prior to the mothers’ narratives being influenced by professional discourses on trauma. In order to address this issue, a two-part process was adopted with mothers initially asked only for consent to record the session and then at the end of the interview whether they consented for the recording to be used as part of the study. It was anticipated that at this stage, the mother would feel less anxious and potentially better able to make an informed, autonomous decision. Concomitant with this process the researchers did not limit attendance at the initial interview.

The first author’s dual relationship (researcher and trauma therapist) presents the classic insider researcher dilemma (Van Heugten 2004; Moore 2007; Greene 2014). It is widely accepted that, *“..what stories we are told, how they are relayed to us, and the narratives that we form and share with others are inevitably influenced by our position and experiences as a researcher in relation to our participants”* (Greene 2014, p. 1). This positioning was made explicit in the participant information and discussed during the consent process. The fact that the Narrative Interview took place before the start of the therapy process and its congruence with aspects of first therapy session assessment seemed to ease this ethical concern.

The duty of care to people talking about sensitive matters was attended to by the normal processes of the Trauma Centre. The interviews were conducted by an experienced therapist who continued the therapeutic work with the families after the assessment interview (whether or not they participated in the study) and additional support could be accessed from the Centre if required. The potential to increase distress or ‘re-trigger’ trauma responses of any individual family member in attendance was assessed as low given the therapeutic context and the congruence of the research question with normal assessment themes. The potential benefit for families of developing a coherent narrative of their experience (Crossley 2000) and the desire of many survivors to contribute to the provision of more effective interventions for future victims/survivors was weighted against this limited risk.

## Results

Brief details of the nature and timing of the traumatic events represented by the ten study cases and the presence of any prior history of adversity are presented in Table 1.

**Fig. 1:**
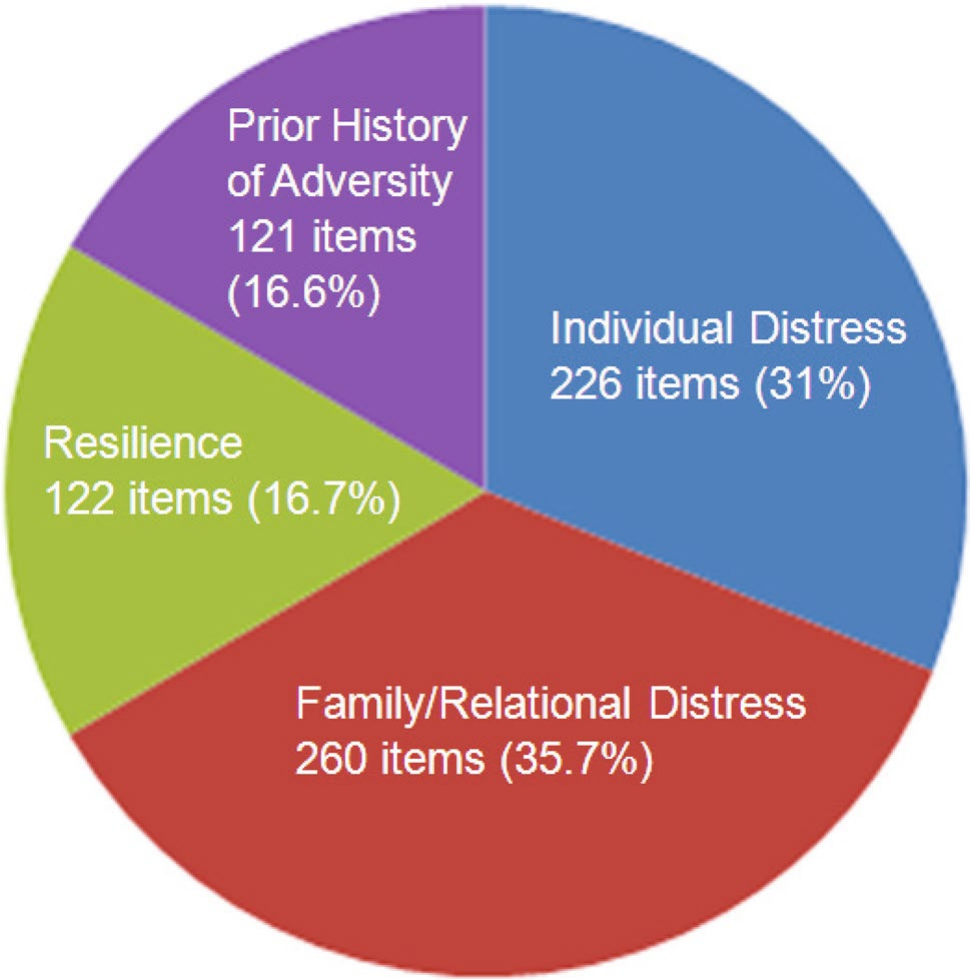
Supra-themes

The traumatic events which precipitated the referral for therapeutic support included physical assault on a young person/family member; sexual assault on a young person; witnessed attack/threat with firearm on family member; sibling suicide; young person overdose and self harm in the context of previous family member suicide. The age of the referred child/young person ranged from 9 to 21 years, seven female and three male. All ten of the families were White—British or Irish, with one from the Irish Traveller community. In cases where there had been a specific event, these had taken place between 2 months and 2 years previously (Fig. 1).

Of the total 729 relevant content items identified for the 10 cases, the headline breakdown of supra-themes is as follows: 35.7% (260 items) represented aspects of ‘Family/Relational Distress’; 31.0% (226 items) were associated with ‘Individual Distress’; 16.7% (122 items) related to dimensions of ‘Resilience’ and 16.6% (121 items) related to ‘Prior History of Adversity’.

### Family/Relational Distress

**Fig. 2:**
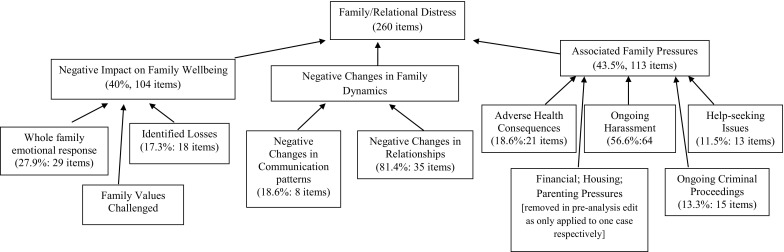
‘Family/relational distress’ framework

The largest supra-theme ‘Family/Relational Distress’ was composed of the following themes; ‘Associated Family Pressures’ (43.5%, 113 items), ‘Negative Impact on Family Wellbeing’ (40.0%, 104 items) and ‘Negative Changes in Family Dynamics’ (16.5%, 43 items) (Fig. 2).

The ‘Associated Family Pressures’ theme incorporates reported deleterious consequences of the traumatic event on the whole family, rather than the direct impact of the event itself. These included ‘Adverse Health Consequences’, ‘Ongoing Harassment’ by the perpetrator, ‘Help-seeking Issues’ and the stress of ‘Ongoing Criminal Proceedings’. The themes of ‘Financial Pressures’, ‘Housing Pressures’ and ‘Parenting Pressures’ were removed from the final data set as these themes applied to one case respectively. Over half of this theme (56.6%; 64 items) related to ongoing harassment from the perpetrator and their social networks indicating significant ongoing deleterious impact in the three cases where this was a feature. A clear sense of ongoing insecurity was articulated by the mother in Case 9 who reported intimidation by a neighbour who had been involved in the physical assault of her partner: *‘my washing machine and tumble dryer’s out in the backyard and when I go to do a wash… I’m scampering out, putting on the washing and you just feel like he’s [perpetrator] looking down’*.

The theme ‘Negative Impact on Family Wellbeing’ includes ‘Identified Losses’, ‘Family Values Challenged’, and descriptions of ‘Whole Family Emotional Response’. In the ‘Whole Family Emotional Response’ theme feelings or emotional states were often ascribed to individuals and the whole family using the same adjectives or phrases: *‘she [daughter] gets nervous… the children were nervous… it is our house is just nervous’* (Case 1). In Case 7 where there was an ongoing threat to the father, the mother noted *‘…they [the children] don’t go to bed without me. If the door knocks, I would jump out of my skin… everybody’s jumping out of their skin’*. Changes in parent–child relationships were noted by a number of mothers as significant losses associated with the traumatic event: *‘I feel close to her but she doesn’t feel close to me anymore’* (Case 6). The theme of ‘Family Values Challenged’ was strongly represented in Case 10 whose teenage son had been the victim of a sectarian assault 6 months previously leaving him badly injured. The mother in this case spoke of her struggle in the aftermath of this unprovoked event of wanting her son to stand-up for himself while not wanting him become violent: *‘he’s [son] a big softy, he’s a big BFG [big friendly giant]… there’s no threatening behaviour in him… I wouldn’t like him to be more violent but I would like for him to be a bit more aggressive’*.

‘Negative Impact on Family Dynamics’ combines ‘Negative Changes in Communication Patterns’ and ‘Negative Changes in Relationships’ and was illustrated by a wide range of changes in family dynamics. For example, the mother in Case 5 whose teenage son had been suffered a physical assault in school leaving him with facial injuries noted the impact on sibling communication and dynamics, remarking how his protective older brothers who had previously engaged in *‘banter’* and *‘horse play’* with their brother, now *‘tiptoed’* around him, unsure how to behave in his presence.

### Individual Distress

**Fig. 3:**
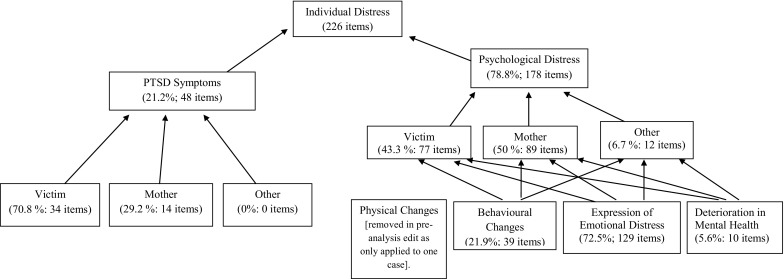
‘Individual distress’ framework

The ‘Individual Distress’ supra-theme was a composite of two major themes ‘PTSD Symptoms’ (as defined by the Post-traumatic Checklist) and non-pathological ‘Psychological Distress’ in the victim, the mother or other family member respectively. The non-pathological ‘Psychological Distress’ theme combined ‘Physical Changes’, ‘Behavioural Changes’, ‘Expressions of Emotional Distress’ and ‘Deterioration in Mental Health’ post trauma (Fig. 3).

Non-pathological ‘Psychological Distress’ was overwhelmingly the dominant contributing theme within the ‘Individual Distress’ supra-theme (78.8%, 178 items) with ‘Expression of Emotional Distress’ the major sub-theme (72.5%), followed by ‘Behavioural Changes’ (21.9%) and ‘Deterioration in Mental Health’ (5.6%). The ‘Physical Changes’ sub-theme applied to only one case and was thus removed in the pre-analysis data edit. The ‘PTSD Symptoms’ theme accounted for 21.2% of the total items identified in this supra-theme.

The contribution of mothers’ responses to the ‘Individual Distress’ supra-theme, inclusive of ‘PTSD Symptoms’ and ‘Psychological Distress’ (89 + 14 = 103 items) was very similar to that reported for the primary victims (77 + 34 = 111 items). However, the balance between ‘PTSD Symptoms’ and ‘Psychological Distress’ was somewhat different with mothers reporting more than twice as many ‘PTSD Symptoms’ for the primary victim compared to their own (34 versus 14 items). Overall each mother’s expression of their own ‘Psychological Distress’ was slightly higher than for their children (89 items to 77).

By far the most dominant theme in mothers’ narratives of personal ‘Psychological Distress’ was the sub-theme ‘Expression of Emotional Distress’ constituting 73 of the 89 content items. A number of mothers reported how the traumatic event had deeply affected their sense of self, described by one mother as *‘I don’t think I am the person I was’* (Case 9). Another expanded her sense of having become a *‘completely different person… I didn’t worry before… it’s not me you know… I’m used to being the strong person… but now other people have to be strong for me’* (Case 4).

A range of emotional responses were described for the victim, mother and other family members. These included tearful distress; worry for the victim, other family members and the future; fear; loss of confidence; loss of control; self-hatred; and anger. Some mothers noted changes in their child’s mood and behaviour which would seem to indicate PTSD symptomatology: *‘Yeah, because she always…. She was always carefree and happy no matter what…laughed and singing, dancing. And now, she’s just moody and grumpy’* (Case 6).

### Resilience

**Fig. 4:**
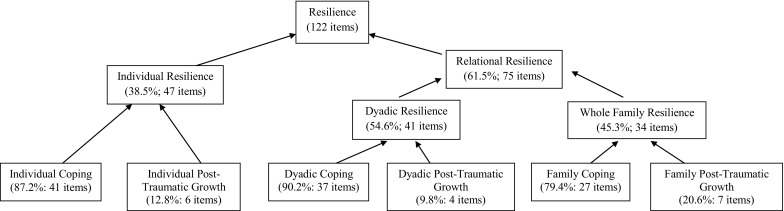
‘Resilience’ framework

Review of the ‘Resilience’ supra-theme showed the ‘Relational Resilience’ theme accounted for 61.5% (75 items) with ‘Individual Resilience’ representing 38.5% (47 items). The ‘Relational Resilience’ theme combined ‘Dyadic Resilience’ (54.6%, 41 items) and ‘Whole Family Resilience’ (45.3%, 34 items). The individual, dyadic and family ‘Post-Traumatic Growth’ components of this supra-theme were a relatively minor contributor with a total of 13.9% (6 + 4 + 7 = 17 items), compared to 86.1% (41 + 37 + 27 = 105 items) for the individual, dyadic and family ‘Coping’ categories (i.e. getting back to pre-trauma levels of functioning) (Fig. 4).

Individual ‘Coping’ was expressed using terms such as ‘soldiering on’ and ‘getting on with it’. As the mother in Case 9 described: *‘I don’t know... I’m having to soldier on… and then I come today and I cried, but I just sort of get on with it, get up in the morning and just go for it.’* ‘Coping’ was also expressed through hope for the future: *‘I want things better for us and also want things better for her. So when she grows up, she is able to deal with stuff’* (Case 6).

There were many examples of dyadic and family ‘Coping’ with family members adjusting their communication and behaviour in ways intended to support the wellbeing of the victim and each other. The mother in case 5 noted a gendered and dyadic component to parental coping, reporting how she and her husband showed their emotion and demonstrated coping in synchronised ways: *‘it is hard and I’ve noticed too. It can be hard on [father] because he’s a daddy… I know [father] is very strong and sometimes he doesn’t show the kids sort of how much it has affected him. I see it, but he’ll be strong for them. I would see the different side*—*he would let his emotion show to me*—*but he tries his best to always let the kids know that he’s their strength… I cry but he can be the strong one.’*

‘Family Post-traumatic Growth’ was well articulated by one mother who spoke of how family relationships and communication had been enhanced when she said, *‘…well, it has made us want to talk more about problems. We talk about everything. You know, it doesn’t matter what it is. We talk about it. It’s made us more closer. It’s made us more protective of each other’* (Case 8).

### Prior History of Adversity

The narratives of 5 of the 10 mothers interviewed (Cases 2, 4, 6, 7, 8) contained reports of prior adverse life events that comprised 30.3% of their content items. Initial referral information indicated that three additional mothers had adverse experiences in their histories that they did not include in their interview narratives (see Table 1). Previous traumatic experiences reported included multiple suicide attempts of another child, death by suicide of close family members, malicious fire setting, sexual assault and domestic violence. Out of the eight cases in which we were aware of a prior history of adversity, it is noteworthy that six included a completed suicide of a significant family member and one of multiple suicide attempts of a family member. For those mothers who spoke of prior adversity, their narratives often contained heart-wrenching tales of multiple difficulties of grave complexity stretching over a number of years. These events seemed to provide the context for the mother’s capacity/struggle to make sense of the current traumatic incident. The mother in Case 2 spoke spontaneously of numerous adverse life events and of how current circumstances were *‘bringing up a lot of memories’*. In addition to the high frequency of reported suicide in this study, a number of mothers also spoke of the experience of domestic violence, often witnessed by the children and reported as only one of a series of adverse events.

## Discussion

The results of this pilot study support the first author’s clinical experience that PTSD symptoms are very much a minority theme (6.6% of total content) in mothers’ spontaneous narratives of trauma impact and they co-exist among a wide variety of responses spanning both negative and positive effects at an individual and relational level. This suggests that PTSD symptoms alone do not represent the full complexity of the phenomenological experience or concerns about the impact of traumatic events on survivors and their families articulated by mothers post trauma.

The prevalence of psychological distress as compared with PTSD symptoms (78.8–21.2%) within the ‘Individual Distress’ supra-theme would indicate that for general clinical cases the concept of psychological distress should be the primary (less pathologising) concept to bring to an understanding of the impact of trauma on individuals, both victims/survivors (including children and young people) and significant others. The argument that the victims/survivors may have recounted more PTSD symptoms if they had narrated their own experience is offset by the evidence supporting the reliability of mothers’ accounts of their children’s wellbeing post-trauma and, if anything, their tendency to over-report children’s distress as their own emotional distress increases (Najman et al. 2001).

The high level of maternal ‘Expression of Emotional Distress’ (12.2% of their total narratives) is a risk factor for child and family wellbeing. The position of mothers, with the gendered cultural primary responsibility for care-giving in many societies, may account for this pronounced distress (Sayer 2005). It may however also have been contributed to by the specific nature of the SQUIN question and the therapeutic context of the interviews. Given that maternal wellbeing is known to be interrelated to and significantly influence the child’s capacity for coping and resilience (Deblinger et al. 2001), study findings affirm the importance of paying close attention to mothers’ wellbeing post-trauma. It is also noteworthy that ‘Family/Relational Distress’ accounts for a greater proportion of mothers’ post-trauma narratives than ‘Individual Distress’ (35.7–31%). In trauma circumstances the potential benefit of family interventions seems axiomatic and in keeping with Saltzman et al.’s findings (2009) about the importance of supporting the synchronicity of family members’ responses as central to the individual and family’s ultimate recovery.

In this study post-traumatic growth constituted a relatively low percentage of the total content items within the Resilience supra-theme (13.9%). One might speculate whether the prevalence of a history of significant adversity in 8 of the 10 families in this study and the impact of high levels of ‘Associated Family Pressures’ constrained the potential for post-traumatic growth.

The evident desire of mothers to spontaneously speak at considerable length about prior difficulties in a first clinical interview gives some indication that these adverse experiences are perceived by mothers to be related to the overall impact of the presenting traumatic event on the individual wellbeing of both themselves, their children and the whole family, and suggests the importance of exploring family experience over time. Such cases highlight the complexity of the impact of traumatic events, which on the surface might appear single events, but in reality have an enduring currency.

Finally, the presence of seven completed suicides (adult and child) and one case of multiple suicide attempts in this study is striking and cannot go without comment. Of course, it could simply be an aberration due to the small sample size but given the importance of the issue it warrants further exploration. There is evidence of a familial dimension to suicide. Brent and Mann (2005, p. 13) state, *‘Both completed and attempted suicide form part of the clinical phenotype that is familially transmitted, as rates of suicide attempt are elevated in the family members of suicide completers, and completion rates are elevated in the family members of attempters.’* It is also well established that early childhood adversity (particularly multiple adverse experiences) is an important risk factor for suicide (Devaney et al. 2012). It is possible that a double vulnerability affects these families. This potential double vulnerability, and the attendant risks of completed suicide, suggest the importance of screening for suicidal ideation/behaviours in families presenting to clinical settings post trauma.

### Study Limitations

The small sample size and the diversity of the presenting traumatic event(s) are key limitations which inevitably constrain the degree to which study results can be generalised. That said, the heterogeneity of traumatic events included in this study is representative of the diversity of presenting issues and wider contexts referred to specialist clinical services.

The results cannot be assumed to reflect the parallel narratives of fathers, other adult caregivers or indeed the children and young people themselves, or the narratives of non-clinical counterparts. The authors are planning to extend this stream of research by conducting a parallel study on the narratives of a clinical sample of children and young people post trauma.

The fact that 8 of the 10 mothers’ narratives were recorded in the presence of family members is an additional confounding factor, although the concern that they may be less likely to report their own distress or PTSD symptoms so as not to upset other family members does not appear to have been an issue given the significant prevalence of maternal distress actually reported.

The assumption of a linear relationship between the number of times something was mentioned by the mother and its significance to them could be incorrect. The counter argument is that minimally articulated or even unspoken themes (in an initial interview) may be the most important or distressing for the client.

## Conclusion

The results of this small pilot study provide insight into the multifaceted nature of trauma responses in families seeking professional help, raising questions regarding the dominance of the PTSD construct in professional therapeutic discourses on psychological trauma and the resultant models of available professional intervention. The restricted prevalence of PTSD symptomology in mothers’ impact narratives indicates that the PTSD construct does not adequately represent the broad ‘lived’ experience of the impact of trauma on survivors’ wellbeing and family relationships. The significantly greater prevalence of non-pathological psychological distress and family/relational distress in mothers’ narratives suggests a broader range of constructs and interventions is required to understand and effectively address the impact of trauma. This is counter to the dominant discourse in many service provision and commissioning contexts, that the clinical needs of trauma survivors are adequately met by cognitive behavioural interventions for individuals with a diagnosis of PTSD. The findings of this study suggest that a broader palette of interventions is required to maximise the recovery potential of individuals and their families following the experience of psychological trauma. In the context of children and young people’s trauma experience, specific support for their primary caregiver would appear to offer benefit to the individual child/young person and the whole family.

The interwoven nature of individual and relational distress, at both a dyadic (couple and parental) and whole family level, in conjunction with awareness of the critical importance of social support for post-trauma wellbeing (Saltzman et al. 2009), supports the contention that Systemic Psychotherapy with its focus on strengthening relationships and interactional processes with significant others as a means to support individual recovery and family resilience (Walsh 2016) would appear to have a valuable contribution to make to trauma recovery.
